# Thank you to our Reviewers

**DOI:** 10.1002/cfg.504

**Published:** 2005


					Comparative and Functional Genomics
Comp Funct Genom 2005; 6: 410-411.
Published online in Wiley InterScience (www.interscience.wiley.com). DOI: 10.1002/cfg.504
Thank you to our Reviewers
The Editor-in-Chief, Section Editors and Editorial Office of Comparative and Functional Genomics
gratefully acknowledge the cooperation of the following scientists in promptly reviewing manuscripts
for the journal.
Tatsuya Akutsu
Mark Albertella
Aileen Allsop
Michael Anderson
Siv Andersson
Miguel Andrade
Rolf Apweiler
Alison Ashcroft
Linda Ashworth
Terri Attwood
Simon Avery
Jurg Bahler
Mike Barnes
Gregory Barsh
Bonnie Bartel
John Belisle
Jeffrey Bennetzen
Joanna Betts
Rishikesh Bhalerao
Pierre-Alain Binz
Alain Blanchard
Jef Boeke
Ulla Bonas
Michael Brand
Andy Brass
Jay Brewster
Jim Bristow
Tony Brooks
Alastair Brown
John Brown
Richard Bruskiewich
Malcolm Buckle
Martha L. Bulyk
Howard Bussey
Rita Casadio
Helen Causton
Stanislaw Cebrat
John Celenza
John-Marc Chandoni
Peter WS Chang
Michael Cherry
George Coghill
Stewart Cole
M. Joseph Colston
Fabrice Confalonieri
Terrance Cooper
Brendan Cormack
Maria Costanzo
Damian Counsell
Neil Curson
Yang Da
Thomas Dandekar
Christoper Danpure
Hidde de Jong
Jeffrey Dean
Tim Dexter
Steve DiFazio
Joaquin Dopazo
Ed Dougherty
Thomas Dougherty
Tilman Drueke
Iain Drummond
Ian Dunham
Arne Elofsson
Grigori N. Enikolopov
Anton Enright
Chris Franklin
Andrew Fraser
Francoise Foury
Teryl Frey
Marcus Frohme
Helmut Fuchs
Terry Gaasterland
Dean Gabriel
Andrzej Galat
Brandon Gaut
Debashis Ghosh
Gerry Graham
Ian Graham
Michael Gray
Marion Greaser
Michael Gribskov
Andrei Grigoriev
Sean Grimmond
Darlene Goldstein
Stephen Gordon
Crisanto Gutierrez
Herwig O. Gutzeit
Udo Hahn
John Hancock
Steven D. Hanes
Andy Hayes
Andreas Heger
Henning Hermjakob
Richard Herrmann
Emma Hill
Karsten Hokamp
Arne Holmgren
Marit Holden
Christine Hoogland
David Hoyle
Martijn Huynen
Joseph Icenogle
Jon Ison
Derek Jamieson
Jean-Luc Jestin
Peter Jongeneel
Gavin Kelsey
Ross King
Nancy Kleckner
Alexey Kondrashov
Eugene Koonin
David Kreil
Anuj Kumar
Vladimir Kuznetsov
Mark Lambrecht
Vincent Laudet
Dariusz Leszczynski
Peter Lichter
Wolfram Liebermeister
Copyright  2006 John Wiley & Sons, Ltd.
Thank you to our Reviewers 411
Gerard London
Ed Louis
Alberto Macario
Carlos Martin
Steve Massy
John McClure
Hayes McDonald
Max Mergeay
Crispin Miller
Todd Mockler
Norman Morrison
Brian Morton
Andy Mungall
Hiroyuki Nakahara
David Ogrydziak
Shuichi Onami
Ian Paulsen
Julian Parkhill
Charles Penn
Graziano Pesole
Jo Peters
Jure Piskur
Andrew Plaut
Stanley Plotkin
Laura Popolo
Tom E. Porter
Richard Poulsom
Rolf Prade
Isak S. Pretorius
Cristin Print
John Quackenbush
Neil Rawlings
Wolf Reik
Eduardo Rocha
Eitan Rubin
Hector Sanchez-Villeda
Chris Sanderson
Manuel Santos
Thomas Schilling
Thomas Schlitt
Steffen Schultze-Kremer
Laurent Segalat
Jude Shavlik
Sudhir Sinha
Katherine Smart
Jacqueline Smith
Michael Snyder
Ed Southern
Lubomira Stateva
Dov Stekel
Derek Stemple
Robert Stevens
Lloyd Sumner
Henk Tabak
Howard Takiff
Chris Taylor
Ulrich Tepass
Johan Thevelein
Chris Town
Peter Tribble
Olga Troyanskaya
Charles Tyler
Peter Uetz
Agnes Ullmann
Alfonso Valencia
Marc Vidal
Alain Vignal
Jaak Vilo
Veronique Vincent
Martin Vingron
Guido Volckaert
Herman Von Tilbeurgh
Marian Walhout
Matt Wayland
Scott Weaver
Jennifer Weller
Lodewyk Wessels
Michael White
Emma Whitelaw
Stefan Wiemann
Gary Williams
Michael Wise
Ernst Wit
Olaf Wolkenhauer
Valerie Wood
Itai Yanai
Brian Yandell
John Yates
Nianshu Zhang
Copyright  2006 John Wiley & Sons, Ltd. Comp Funct Genom 2005; 6: 410-411.

				

